# Transporter Engineering in Microbial Cell Factory Boosts Biomanufacturing Capacity

**DOI:** 10.34133/2022/9871087

**Published:** 2022-06-15

**Authors:** Xiaodong Lv, Haijie Xue, Lei Qin, Chun Li

**Affiliations:** ^1^Key Laboratory of Medical Molecule Science and Pharmaceutics Engineering, Ministry of Industry and Information Technology, Institute of Biochemical Engineering, School of Chemistry and Chemical Engineering, Beijing Institute of Technology, Beijing, China; ^2^Key Lab for Industrial Biocatalysis, Ministry of Education, Department of Chemical Engineering, Tsinghua University, Beijing, China; ^3^Center for Synthetic and Systems Biology, Department of Chemical Engineering, Tsinghua University, Beijing, China

## Abstract

Microbial cell factories (MCFs) are typical and widely used platforms in biomanufacturing for designing and constructing synthesis pathways of target compounds in microorganisms. In MCFs, transporter engineering is especially significant for improving the biomanufacturing efficiency and capacity through enhancing substrate absorption, promoting intracellular mass transfer of intermediate metabolites, and improving transmembrane export of target products. This review discusses the current methods and strategies of mining and characterizing suitable transporters and presents the cases of transporter engineering in the production of various chemicals in MCFs.

## 1. Introduction

Biomanufacturing uses renewable biomass to produce bioenergy, biomaterials, natural products, and bulk chemicals, which has important significance for carbon emission reduction and sustainable development [1, 2]. Microbial cell factories (MCFs), as the core of biomanufacturing, are generally manipulated via metabolic engineering and synthetic biology techniques for producing diverse compounds. At present, many strategies have been used to improve the efficiency and capacity of MCFs, including enhancing pathway flux [3], inhibiting competitive pathways [4], cofactor engineering [5], and enzyme engineering [6]. In particular, since MCFs are regarded as “production workshops,” the mass transfer efficiency among the “production units (cells or organelles)” was usually insufficient, especially for eukaryotes, which extremely limits the further improvement of MCFs.

To solve this problem, transporter engineering was provided as an alternative strategy that can enhance substrate absorption, promote the intracellular mass transfer of intermediate metabolites, and improve the transmembrane export of target products. Generally, several strategies have been used for mass transfer intensification in MCFs. Subcellular compartmentalization strategy have been adopted to strengthen metabolic mass transfer by introducing a series of reactions into one compartment/organelle, which improved local concentrations in space for increasing product concentration [7–20]. Membrane engineering was also an effective strategy in the export of hydrophobic products by modifying the cell membrane structure genetically [21–23] or adding exogenous reagents such as cyclodextrins [24, 25], dodecane [26], and olive oil [27], which significantly released intracellular storage space and released product inhibition. Mining, expressing, and remolding transporters of target compounds (i.e., transporter engineering) is the most direct way to import or export a specific substrate. Transporter engineering has received more and more attention due to its specificity, efficiency, and simplicity.

Herein, we summarized the current methods and strategies for mining and characterizing suitable transporters and introduced cases for improving the manufacturing efficiency of MCFs through transporter engineering. The understanding of cellular transport process and the application of transporter engineering would provide novel insights into the construction of MCFs for the biomanufacturing process.

## 2. Mining and Characterizing Suitable Transporters

At present, the reported information about transporters is still inadequate, which causes tremendous challenge for engineering molecular transport in MCFs. Therefore, it has become exceptionally crucial for mining and characterizing more specific transporters for the target compounds (Table 1).

**Table 1:** Transporter applications in microbial cell factories.

Transporter	Species	Compound	Function	Reference
*Substrates*				
Gal2p	*S. cerevisiae*	Xylose	Improved the transport rate and accelerated utilization of xylose.	[40]
AraT	*S. cerevisiae*	L-arabinose	Transported L-arabinose with high specificity and high affinity.	[36]
XylE	*P. putida*	Xylose	Broadened metabolic capacity towards new substrates.	[43]
GatA	*S. cerevisiae*	D-galacturonic acid	Achieved coutilization of D-galUA and D-glucose.	[44]
Lac12	*S. cerevisiae*	Lactose	Increased uptake of the lactose.	[66–68]
*Intermediate metabolites*				
ShiA	*E. coli*	3-Dehydroshikimate	Enhanced reuptake of intermediate metabolite from extracellular to cytoplasm.	[45]
FadL	*E. coli*	Palmitate	Achieved reuptake of excreted intermediate metabolite.	[69]
*Δ*pxa1	*S. cerevisiae*	Fatty acyl-CoA	Increased production of fatty acyl-CoA in the cytoplasm.	[46]
NtJAT1, NtMATE2	*S. cerevisiae*	Tropine	Alleviated vacuolar intermediate metabolite transport limitations.	[47]
*Target products*				
AcrE, MdtE, MdtC	*E. coli*	Medium-chain fatty acid	Increased extracellular MCFA concentration by 59.7%, 43.2%, and 83.1%.	[55]
FATP1	*S. cerevisiae*	Fatty alcohol	Enabled an increased cell fitness for fatty alcohol production.	[56]
FATP1	*S. cerevisiae*	1-Alkenes	Improved the extracellular and total 1-alkene production.	[57]
MacA, TolC, MacB	*E. coli*	6-Deoxyerythronolide B	Increased the 6dEB titers.	[60]
Orf14, Orf3	*Burkholderia*	Epothilones	Raised the ratio of extracellular to intracellular accumulation from 9.3 : 1 to 13.7 : 1.	[62]
TolC, AcrB	*E. coli*	amorphadiene	Increased yield by 46%.	[59]
AcrA, TolC AcrB,	*E. coli*	Kaurene	Increased yield by 82%.	[59]
Snq2p	*S. cerevisiae*	*β*-Carotene	Improved *β*-carotene secretion level by 4.04-fold.	[64]
AcrAB	*E. coli*	Limonene	Reduced limonene toxicity.	[70]
Bfr1	*S. cerevisiae*	Caffeine	Enhanced cellular resistance to caffeine.	[34]
AbPUP1, AbLP1	*S. cerevisiae*	Littorine and hyoscyamine	Exported vacuolar littorine and hyoscyamine to the yeast cytosol.	[37]
AtDTX1	*E. coli*	Reticuline	Achieved the secretion of high levels of reticuline.	[65]
MttA	*A. niger*	*cis*-aconitic acid	Secreted 9.8 g/L aconitic acid after 240 h of cultivation.	[71]
Spmae∗	*S. cerevisiae*	L-malic acid	Increased the accumulation.	[50]
AtABCG29	*S. cerevisiae*	Coumaryl alcohol	Increased cellular tolerance to *p*-coumaryl alcohol.	[72]
PtPTP	*Phaeodactylum tricornutum*	Pyruvate	Enhanced biomass, lipid contents, and growth.	[73]
DCT1	*A. niger*	Malic acid	Improved malic acid production by 36.8%.	[74]
MTT	*Y. lipolytica*	Itaconic acid	Enhanced itaconic acid titer by 10.5-folds.	[75]
RibM	*B. subtilis*	Riboflavin and roseoflavin	Increased the production of riboflavin and roseoflavin.	[76]
PP_1271	*P. putida*	Propionic acid	Improved cellular tolerance to PA.	[48]
YbjE	*Synechococcus sp*	Lysine	Generated a large pool of lysine in the extracellular media.	[77]
Qdr3	*S. cerevisiae*	Muconic acid	Increased cellular tolerance to glutaric, adipic, muconic, and glutaconic acid.	[78]
CexA	*A. niger*	Citric acid	Enhanced the secretion of citric acid.	[79]
M2	*E. coli*	Proton	Increased acid tolerance.	[80]
SerE	*C. glutamicum*	L-serine	Increased L-serine efflux.	[51]
Tpo2p	*S. cerevisiae*	cis,cis-muconic acid, protocatechuic acid	Improved the production of target compound.	[39]

**Figure 1:**
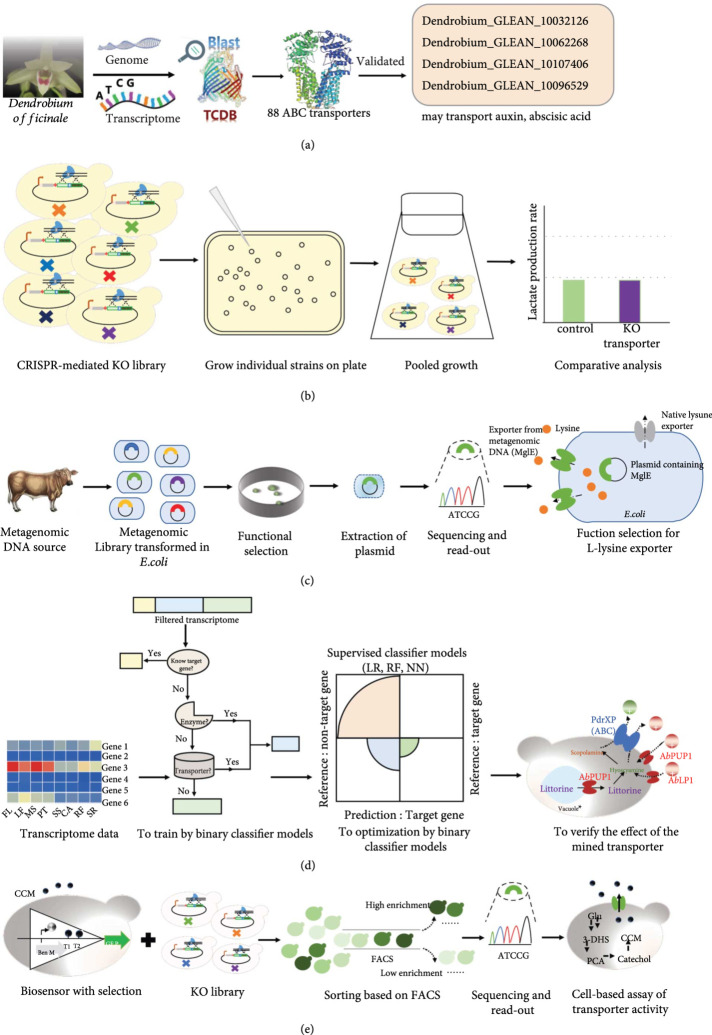
Methods for mining and characterizing suitable transporters: (a) predicting transporter via bioinformatics database; (b) screening natural transporters by establishing knock-out library; (c) identifying exporters by constructing and screening a metagenomic library; (d) mining transporters from the transcriptome by training, optimizing, and testing binary classifier models; and (e) high-throughput screening of transporters based on biosensors.

Bioinformatic tools are becoming popular due to their ability to analyze huge amounts of biological data [28]. AntiSMASH is a powerful tool for identifying gene clusters, which can annotate information about transporters [29]. Transporter classification database (TCDB; http://www.tcdb.org) is a common and freely accessible reference database [30]. Generally, the same transporter family can recognize similar substrate structures. Therefore, researchers have identified 88 ABC transporters from *Dendrobium officinale* via sequence alignment from TCDB [31], and four transporters were predicted for the transportation of abscisic acid and auxin by transcriptomic comparison [32] (Figure 1(a)).

The function of natural transporters can be identified by gene knockout. However, the efficiency of this method was usually not obvious due to the redundancy of intracellular transporters and the complex network of their interactions. For instance, after knocking out the aqua (glycero) porin family and all known carboxylic acid transporters using CRISPR-Cas9 in *Saccharomyces cerevisiae,* the extracellular lactate production rate remained unchanged, indicating there existed some unknown transporters or mechanisms to export lactate (Figure 1(b)) [33]. Alternatively, constructing the mutation library of transporters was shown to be effective for high-throughput mining and characterizing the desired transporter. To export caffeine and relieve its toxicity to yeast, a mutation library of endogenous ABC-transporter brf1 was constructed, from which a mutant was screened out to increase caffeine resistance [34]. To find an efficient L-lysine export system, a metagenomic library of cow dung samples was constructed. After plating recombinants on high L-lysine concentration media, a novel lysine efflux transporter mglE was screened out, which improved the L-lysine tolerance of *Escherichia coli* by 40% and increased the L-lysine productivity of *Corynebacterium glutamicum* by 12% (Figure 1(c)) [35].

System biology and machine learning provided new approaches for mining transporter genes. For example, through analyzing transcriptomic data of *Penicillium chrysogenum* in D-glucose and L-arabinose restricted culture, respectively, the fungal transporter PcAraT specifically transporting L-arabinose instead of xylose and glucose was identified [36]. The transcriptomic data of *Atropa belladonna* were analyzed through binary classifier supervised learning models (logical regression, random forest, and feedforward neural network). The supervised classifier models based on tissue description showed greater efficacy in predicting transporters than traditional regression- and clustering-based methods. As a result, two identified transporters, AbPUP1 and AbLP1, were found to increase the production of target alkaloids in the engineered yeast (Figure 1(d)) [37].

Genetically encoded biosensors [38] are another powerful tool for high-throughput screening of strains producing or transporting target compounds. Researchers constructed a knockout library of 361 nonessential native transporters in *S. cerevisiae* via CRISPR/Cas9 followed by fluorescence-activated cell sorting (FACS) based on the biosensor of the target organic acid compounds *cis, cis*-muconic (CCM) and protocatechuic acids (PCA) [39]. As a result, Tpo2 was validated as an importer of CCM and PCA through *Xenopus* expression assays (Figure 1(e)) [39].

## 3. Enhancement of Substrate Absorption by Importer in MCFs

Low substrate uptake rates would hamper the productivity of MCFs, especially in engineered strains that use unnatural substrates (Table 1). Therefore, it is particularly important to improve substrate absorption and enhance transfer efficiency through targeted importer expression in MCFs.

**Figure 2:**
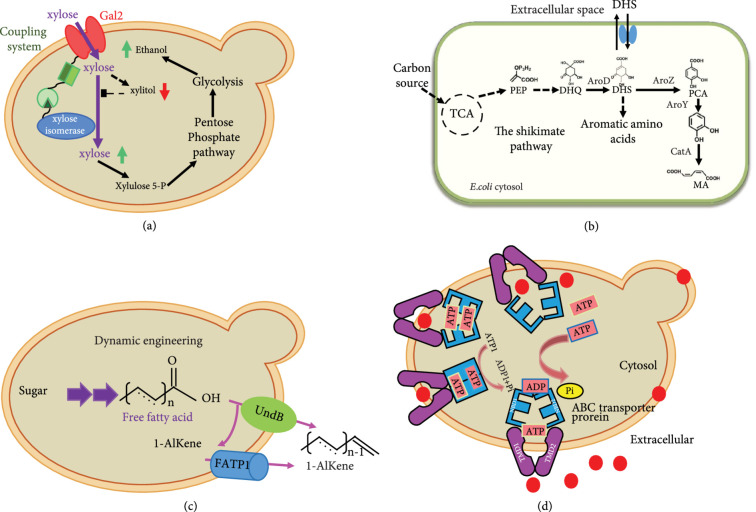
Application of transporter engineering in the construction of MCFs. (a) The Gal2 transporter was coupled with xylose isomerase to improve the xylose transport rate; (b) schematic of the cis, cis-muconic acid biosynthetic pathway and the intermediate DHS transporter; (c) 1-alkene secretion by expressing the long-chain fatty acid transporter FATP1 from *Homo sapiens*; and (d) improvement of the export and production of *β*-carotene by overexpressing endogenous ABC transporters.

Lignocellulose-derived pentose sugars (mainly D-xylose and L-arabinose) are not natural substrates of baker’s yeast. The utilization of pentose can substantially improve bioresource utilization. An artificial complex consisting of endogenous sugar transporter Gal2 and heterologous xylose isomerase (XI) was constructed in *S. cerevisiae*, which significantly improved the substrate uptake rate and simultaneously reduced the production of byproduct xylitol (Figure 2(a)) [40]. The galactose transporter Gal2 and the low-activity hexose transporter Hxt9 could transport L-arabinose with low affinity ($K_{\text{m}}=57\sim 371\;\text{mM}$) [41, 42]. Therefore, a high-affinity ($K_{\text{m}}=0.13\;\text{mM}$) and high-specificity L-arabinose transporter PcAraT from *Penicillium chrysogenum* was identified by characterizing sugar uptake kinetics in *S. cerevisiae*, which contributed to rapid and efficient conversion of L-arabinose [36]. In another study, the absorptivity of oligosaccharides and pentose was reinforced in *Pseudomonas putida* when the native ABC transporter complex PP1015~PP1018 was overexpressed [43]. D-galacturonic acid and D-glucose were coutilized by identifying and expressing a heterologous transporter GatA from *Aspergillus niger*, which realized the production of galactonic acid directly from industrial orange peel waste [44].

## 4. Promotion of the Intracellular Mass Transfer of Intermediate Metabolites

Strengthening the reuptake of intermediate metabolites or the mass transfer between cells and extracellular media or subcellular compartments could significantly improve the flux of metabolic pathways, which is essential for the manufacturing efficiency and capacity of the MCFs. In the *cis*, *cis*-muconic acid production strain, the crucial intermediate 3-dehydroshikimic acid (DHS) can diffuse to the outside of the cell along the concentration gradient, resulting in the draining of precursor. By expressing a membrane-bound transporter ShiA to import DHS into the cytosol, the production of *cis*, *cis*-muconic acid was significantly improved [45] (Figure 2(b)). Acyl-CoA degrades to acetyl-CoA through the peroxisomal *β*-oxidation pathway in *S. ce*revisiae, which limits the production of cytosolic acyl-CoA. Therefore, the fatty acyl-CoA peroxisomal transporter Pxa1 was knocked out to prevent oxidation, which improved the production of acyl-CoA-derived triacylglycerols [46]. Researchers introduced the tobacco-derived multidrug and toxic compound efflux proteins NtJAT1 and NtMATE2 with nicotine transport ability into yeast, which alleviated vacuolar intermediate metabolite transport limitations, eventually increasing the titers of the target alkaloids hyoscyamine and scopolamine by 74% and 18%, respectively [47].

## 5. Improvement of the Transmembrane Export of Target Products in MCFs

Export of target products in MCFs has many benefits, including releasing intracellular space, eliminating product inhibition, and reducing potential product toxicity. Propionic acid is a valuable C3 platform chemical, but it is toxic to microorganisms. Propionic acid tolerance and production in *P. putida* were increased by overexpressing the major facilitator superfamily (MFS) transporter gene cluster PP_1271 [48]. However, the production did not fluctuate greatly after deleting the cluster PP_1271, which showed more than one transporter regulating propionic acid tolerance and confirmed the complexity of the transport mechanism. A C4-dicarboxylate transporter Spmae from *Schizosaccharomyces pombe* was found to export L-malic acid effectively [49]. Researchers found that Spmae can be modified by ubiquitin, which might result in significant degradation. By employing a deubiquitination strategy, the accumulation of L-malic acid was improved in *S. cerevisiae* [50]. In another case, to export L-serine from the cell, a novel exporter SerE was overexpressed, and the titer of L-serine reached 43.9 g/L in *C. glutamicum* combining with the strengthening of L-serine synthetic pathway, which further enhanced its industrial application [51].

The export of many fuel chemicals was conducive to their bioproduction, such as fatty acids [52], fatty alcohols [53, 54], and alkanes [52]. The coexpression of efflux transporters mdtE, acrE, and mdtC in combination with the deletion of the influx transporter cmr increased extracellular medium-chain fatty acids (C6-C10, MCFAs) titer and endowed host strains with more adaptability to harsh environments [55]. In order to alleviate growth inhibition and reduce extraction cost, the export of fatty alcohols was promoted by about fivefold after expressing human fatty acid transporter FATP1 in yeast [56]. FATP1 also facilitated the production and secretion of alkenes according to the similar hydrophobic properties between long-chain fatty acids and alkenes. As a result, more than 80% of alkene was exported, which immensely reduced the cost of downstream extraction and separation and further improved the economics of the process (Figure 2(c)) [57].

Polyketides are a large class of natural products with great therapeutic value. Resistance-nodulation-cell division (RND) family efflux pumps play major roles in the resistance of gram-negative bacteria to a wide range of compounds, such as polyketides [58]. An RND efflux pump typically consists of three different components, an inner membrane protein (e.g., MacB), an outer membrane protein (e.g., TolC), and a periplasmic membrane adapter protein (e.g., MacA), which are organized in a complex structure with a specific ratio [59]. The highest titer of 6-deoxygibberellin B (6dEB, erythromycin precursor) was achieved with the combination of MacA, MacB, and TolC in *E. coli* [60]. It is noteworthy that the improvement was significantly higher than those of expressing all single components of pumps alone. Therefore, the coordinative interaction between pump components is indeed important for transporter engineering. Generally, the expression of RND efflux pumps is often tightly controlled by the relevant regulatory proteins. For example, five transcriptional activators YdeO, MarA, RpoH, EvgA, and Fnr, which are responsible for activating the multidrug efflux pumps, were tested for improving polyketide production [60]. In the treatment of cancer, epothilone is a polyketide compound with a better curative effect and milder side effects than taxane [61]. The ratio of extracellular to intracellular accumulation of epothilone was boosted from 9.3 : 1 to 13.7 : 1 by applying two multidrug efflux pumps, Orf14 and Orf3 in *Burkholderia*, thereby promoting the forward biosynthesis of the heterologous polyketide compound epothilone [62].

Terpenoids are the largest family of secondary metabolites of plants, and they are widely distributed in archaea, bacteria, and eukaryotes [63]. The RND efflux pump was also efficient for the cellular exportation of the sesquiterpene amorphadiene and the diterpene kaurene [59]. Interestingly, the three components of tripartite efflux pumps played varied effect on different compounds. For amorphadiene production, the highest yield was achieved with the combination of TolC and AcrB; the three-component combination AcrA-TolC-AcrB achieved the highest yield of kaurene in *E. coli* [59]. The coordinative interaction between pump components was vital for transporter engineering. The extracellular production of hydrophobic *β*-carotene was enhanced by 4.04-fold through adopting an inducible GAL promoter to overexpress the endogenous plasma membrane ABC transporter Snq2p in *S. cerevisiae* [64] (Figure 2(d)). As an important intermediate compound in the alkaloid synthesis pathway, the yield of reticuline increased by 11-fold in *E. coli* by introducing the multidrug and toxic compound efflux family transporter AtDTX1 from *Arabidopsis thaliana* [65].

## 6. Conclusion and Perspectives

Transporter engineering has been documented to improve substrate absorption, promote the intracellular mass transfer of intermediate metabolites, and reinforce the transmembrane export of target products, which play a decisive role in the metabolism and mass transfer of the MCFs. However, reported information about transporters is insufficient, severely limiting the application of transporter engineering in MCFs. Therefore, it is extremely necessary to vigorously develop efficient methods and strategies for mining and characterizing transporters. The current methods for identifying specific transporters still have limitations, such as relatively low throughput, low efficiency, and labor intensive. It is necessary to develop high throughput, low-cost, and efficient methods for automated identification and characterization of transporters.

Transporters generally have a broad substrate spectrum, which increases the transport flux of target compounds through the synergistic effect of multiple transporters. The heterologous production of various classes of compounds could combine different types of pumps. However, selecting the proper type of pump is also vital for a specific heterologous product. In nature, the expression of some efflux pumps is often tightly controlled by the relevant regulatory proteins. Thus, tuning the expression of pump regulators may be an effective option for transporter engineering as well. As the transport mechanism becomes clear, the semirational or rational design based on the protein structure could further expand their substrate spectrum and improve the transport affinity and transport rate of target compounds. In addition, combining transporter engineering with other regulation strategies may further boost production and efflux of target compounds based on their synergism.

## Data Availability

All data are available in the main text.
